# A Review of Neurofeedback Training for Improving Sport Performance From the Perspective of User Experience

**DOI:** 10.3389/fnins.2021.638369

**Published:** 2021-05-28

**Authors:** Anmin Gong, Feng Gu, Wenya Nan, Yi Qu, Changhao Jiang, Yunfa Fu

**Affiliations:** ^1^School of Information Engineering, Engineering University of People’s Armed Police, Xi’an, China; ^2^Department of Psychology, College of Education, Shanghai Normal University, Shanghai, China; ^3^Key Laboratory of Sports Performance Evaluation and Technical Analysis, Capital Institute of Physical Education, Beijing, China; ^4^School of Automation and Information Engineering, Kunming University of Science and Technology, Kunming, China

**Keywords:** neurofeedback training, brain nerve regulation, sport performance, user experience, electroencephalograph

## Abstract

Neurofeedback training (NFT) is a non-invasive, safe, and effective method of regulating the nerve state of the brain. Presently, NFT is widely used to prevent and rehabilitate brain diseases and improve an individual’s external performance. Among the various NFT methods, NFT to improve sport performance (SP-NFT) has become an important research and application focus worldwide. Several studies have shown that the method is effective in improving brain function and motor control performance. However, appropriate reviews and prospective directions for this technology are lacking. This paper proposes an SP-NFT classification method based on user experience, classifies and discusses various SP-NFT research schemes reported in the existing literature, and reviews the technical principles, application scenarios, and usage characteristics of different SP-NFT schemes. Several key issues in SP-NFT development, including the factors involved in neural mechanisms, scheme selection, learning basis, and experimental implementation, are discussed. Finally, directions for the future development of SP-NFT, including SP-NFT based on other electroencephalograph characteristics, SP-NFT integrated with other technologies, and SP-NFT commercialization, are suggested. These discussions are expected to provide some valuable ideas to researchers in related fields.

## Introduction

In 1961, American psychologist Razran first proposed the concept of biofeedback, suggesting that people can use physiological instruments to observe their physiological changes and learn to control themselves to avoid harmful stimuli (Razran, 1961). Since then, biofeedback has become an important research topic. Among them, neurofeedback training (NFT), which refers to perception and learning of one’s own brain signals, is the most widely used technique in biofeedback. Further, NFT is not only the predecessor and an important part of the brain-computer interface (BCI) technology and an important aspect of neural engineering, but also one of the key technologies of brain–computer intelligent fusion (Jeunet et al., 2018; Baqapuri et al., 2021).

Neurofeedback training, which is a non-invasive, safe, and effective means of regulating the nerve state of the brain, was initially applied to the prevention and rehabilitation of clinical nerve illness or mental illness and then gradually was expanded to help improve healthy individuals’ external performance (Marzbani et al., 2016; Sitaram et al., 2016). Based on the signal modality, NFT includes electroencephalogram (EEG) NFT, functional magnetic resonance imaging (fMRI) NFT, and functional near-infrared spectroscopy (fNIS/fNIRS) NFT, while EEG NFT is one of the most commonly adopted (Watanabe et al., 2017; Trambaiolli et al., 2018).

Researchers use a “top-down” approach to improve external performance by regulating brain function and influencing behavior. For example, using NFT to improve individuals’ cognitive ability can enhance focus and memory, as well as motor ability, helping them achieve better sport performance (Gruzelier, 2014a,b,c). In sports science, improving sport performance has been an important research topic. In contrast to the traditional training of strengthening endurance and speed, NFT mainly focuses on the psychological state of athletes. Some studies have shown that NFT can teach athletes to control their mental state and thus improve their sport performance. Therefore, NFT has been widely studied.

Landers et al. (1991) first utilized NFT to improve sport performance (SP–NFT) with 24 pre-professional archers as participants. Their results showed that the shooting performance of the participants who enhanced the right temporal activity of their brain increased, while that of those who enhanced the left temporal activity decreased (Landers et al., 1991). Similarly, Paul et al. (2011) conducted four weeks of SP–NFT with school-level archers and found that their pre-competition pleasure levels, pre-competition arousal levels, and post-competition arousal levels had increased, and their archery shooting performance also had improved (Paul et al., 2011).

Raymond et al. (2005) found that the use of alpha and theta SP–NFT in Latin dancers’ performance improved dance performance after training (Raymond et al., 2005). Meanwhile, Gruzelier et al. (2014) found that alpha and theta SP–NFT improved dancers’ creativity but without significantly impacting dance performance or anxiety (Gruzelier et al., 2014).

Arns et al. (2008) applied SP–NFT in golf during the preparation stage, finding a 25% decrease in participants’ average score (Arns et al., 2008). In addition, Cheng et al. (2015a) found that SP–NFT significantly improved golf putting performance in mean distance, standard deviation, and successful training ratio (Cheng et al., 2015a). Ring et al. (2015) studied SP–NFT in recreational golfers and found that players learned to reduce their frontal alpha rhythm before hitting, but SP-NFT failed to selectively enhance putting performance (Ring et al., 2015).

Rostami et al. (2012) studied an SP–NFT scheme combining beta and alpha rhythms and found a significantly improved mean of shot results after training when compared with the control group (Rostami et al., 2012). Similarly, Gong et al. (2020) compared the effects of sensorimotor rhythm (SMR) SP–NFT and alpha SP–NFT on rifle shooting, finding that the mean of shot results of amateur shooters involved in SMR SP–NFT had significantly improved, but that with alpha SP-NFT had decreased (Gong et al., 2020).

Faridnia et al. (2012) evaluated the effects of SP–NFT on anxiety reduction in female swimmers, finding that SP–NFT could reduce their competitive trait anxiety (Faridnia et al., 2012). Mikicin et al. (2015) studied the effects of SP–NFT on the cognitive function of 35 college athletes (swimming, fencing, judo) and found an improvement in visual attention response and Kraepelin work curve (Mikicin et al., 2015).

However, these previously mentioned SP–NFT studies differ greatly in experimental paradigms and parameters, and different scholars have different opinions about the efficacy of SP–NFT. For example, Gruzelier et al. (2014) believe that SP–NFT has great potential to improve sport performance and is an effective training method (Gruzelier, 2014a). However, Mirifar et al. (2017) believe that the quality of existing studies can differ, with only a few having used strict double-blind, placebo-control experiments, so their results may not support the effectiveness of SP–NFT (Mirifar et al., 2017).

Xiang et al. (2018) conducted a meta-analysis of previous SP–NFT studies, and their statistical results show that SP–NFT is an effective brain regulation method that could influence motor behavior by changing participants’ EEG characteristics (Xiang et al., 2018). However, because few research samples have met strict standards, the reliability of the results might be in question.

From the perspective of professional technology, these comments provide theoretical support and guidance for the application and development of SP–NFT. However, few studies have considered this treatment from the perspective of the users, including coaches, athletes, and general enthusiasts, which may explain why SP–NFT has seemed less practical in sports training.

Furthermore, existing SP–NFT research paradigms are diverse and vary widely among feedback schemes. Some SP–NFT studies require participants to train in a laboratory, while others require participants to perform their activities in their usual locations. Some SP–NFT studies require participants to remain focused, while others require mental relaxation.

Although the training methods in these studies differ, most studies only use sport performance as the evaluation index. If the evaluation is not properly classified, researchers may obtain incorrect analysis results, and some non-standard SP–NFT results may even affect the evaluation of the effectiveness of the overall SP–NFT research.

To solve the above-mentioned problems, it is necessary to establish a more detailed classification of SP–NFT from the perspective of users. At the same time, we should minimize overly professional SP–NFT descriptions to facilitate coaches and athletes to understand and learn NFT. This can help promote the development of SP–NFT research and its application and make the results more practical and instructive.

Therefore, this article first summarizes NFT’s concept, process, and research methods and then, from the point of view of SP–NFT users, puts forward an SP–NFT classification method based on their experiences. We use this method to comment on the current research, to analyze the key issues in SP–NFT’s development, and finally to look forward to future development directions.

## NFT Processes and Research Methods

**FIGURE 1:**
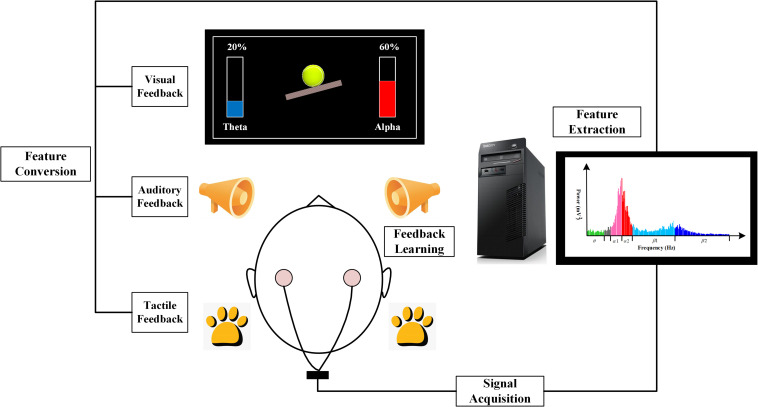
Principle block diagram of electroencephalograph (EEG) neurofeedback training (NFT) system. A typical NFT system usually consists of four stages: (1) signal acquisition, (2) feature extraction, (3) feature conversion, and (4) feedback learning.

As shown in Figure 1, the typical NFT process includes four stages: (1) signal acquisition, (2) feature extraction, (3) feature conversion, and (4) feedback learning. NFT measures an individual’s brain activity using an electronic instrument, and this information is selectively converted into that person’s easily perceived signal. By learning and controlling the feedback signal provided by the instrument, participants can self-regulate their minds so as to attain the goals of their rehabilitation and treatment for specific diseases or for an improved physiological state.

Carrying out a complete NFT research study should usually include three steps: (1) determine the relationship between the sport performance and the brain’s neural mechanism, (2) test the trainability of the NFT, and (3) verify the effects of the NFT.

First, determining the relationship between sport performance and the brain neural mechanism involves revealing the neural mechanism in motor behavior and excavating key neural characteristics that can affect sport performance. For example, some scholars have found that activity inhibition in the left temporal region and activity activation in the right temporal region of the brain during the preparation stage of shooting and archery can lead to improvement (Hatfield et al., 1984; Salazar et al., 1990). Other scholars have found that high sport performance in fine-type movements is usually accompanied by a decrease of frontal midline theta rhythm and an increase of central SMR (Doppelmayr et al., 2008; Cheng et al., 2015b). These results reveal that sport performance is closely related to cerebral nerve activity, so the regulation of these neural activities may, in turn, affect sport performance. These results are the physiological basis for carrying out SP–NFT research.

Second, to test the trainability of an NFT is to test whether participants can successfully learn how to use that NFT, to discuss learning strategies and difficulty of the learning process, to report the proportion of and understand the reasons for non-responders, to determine whether feedback training can change the brain’s “baseline” neural activity, and to explain any problems related to training practice (Cho et al., 2007; Zoefel et al., 2010). Not all neural characteristics are suitable as NFT characteristics, and the training difficulty of each characteristic is different. For example, the training difficulty of regulating the ratio of different frequency band power may be greater than that of the frequency band power of a single-brain region, and the training difficulty of regulating multiple rhythm may be greater than that of regulating a single rhythm (Collura, 2013; Nan et al., 2015).

Meanwhile, many NFT studies have also reported a certain number of non-responders among the participants, accounting for about 20–30% of the total. These cannot regulate their brain activities, and the reasons for this phenomenon are still unclear (Enriquez-Geppert et al., 2014). The neuroplasticity test is also an important part of trainability analysis, which involves checking whether the brain activity changes after NFT. Ghaziri et al. (2013) found significant changes in the gray matter and white matter volume of participants after 40 NFT sessions, which is one of the most direct examples of NFT neuroplasticity (Ghaziri et al., 2013).

Finally, the verification of NFT’s training effects involves whether the behavioral outcome changes significantly due to NFT and whether a significant correlation exists between the NFT learning and the change in behavioral outcome. Similar to other interventions in the medical field, NFT also uses controlled experiments to test the effect of training, which includes the following details (Mirifar et al., 2017; Ros et al., 2020):

(1) All participants should be divided into an experimental and a control group, and no significant difference should exist in all aspects of the conditions between the two groups;
(2) The number of participants in each group should exceed the minimum statistical standard, usually more than 17 per group (Mirifar et al., 2017);
(3) The experimental design should adopt a double-blind or even triple-blind design to ensure that the experimenters and participants do not know the grouping details;
(4) The control group should adopt the placebo-control design, allowing control group participants to believe that they also participate in the real feedback training.

## Classification of SP–NFT Based on User Experience

Previous SP–NFT studies are usually classified by experimental paradigm, such as theta rhythm training, alpha rhythm training, SMR training, or other combination of multiple rhythms (Mirifar et al., 2017; Xiang et al., 2018). This classification method is convenient for researchers to quickly understand the experimental paradigm of feedback training and can facilitate communication between scholars. However, this method is not applicable to non-professional personnel. For example, coaches and athletes may not understand what theta rhythms are, why some training schemes require theta rhythms to be reduced, and why others require increased theta rhythms.

**FIGURE 2:**
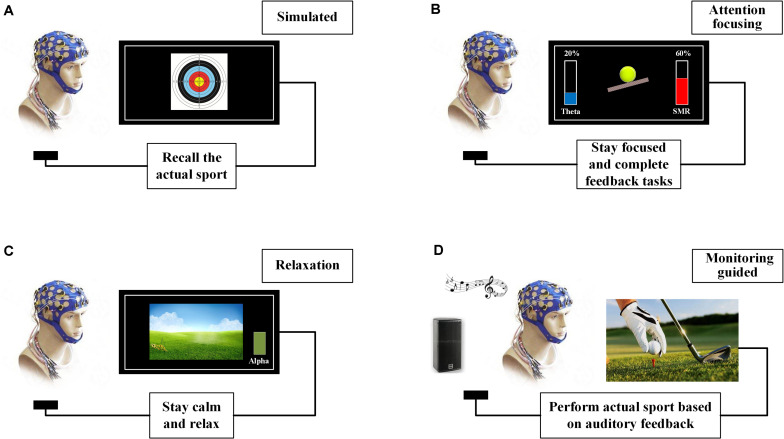
Different types of SP–NFT schemes. **(A)** Simulated training scheme in which participants recall the actual sport process. The feedback feature is the EEG characteristics when athletes recall the best sport performance. **(B)** Attention-focusing training scheme, during which participants remain highly focused. The feedback feature is inhibition theta or enhancement SMR. **(C)** Relaxation training scheme, during which participants maintain a relaxed mind. The feedback feature is increasing theta or inhibition alpha. **(D)** Monitoring-guided training scheme, in which participants directly perform sport behavior at an optimal arousal level 9.

Therefore, this article proposes an SP–NFT scheme classification method based on user experience (Figure 2). From the user’s point of view, the most important training characteristics of SP–NFT are summed up in a way that describes a participant’s experience (Table 1). This method can help increase non-professional individuals’ understanding of SP–NFT and can also facilitate the quantitative and training-oriented evaluation of the SP–NFT training effects.

**TABLE 1:** SP-NFT relevant research literatures.

Author/year	Participants/N	SP-NFT scheme	Indicating the task	Number of sessions	Duration of each session	Type of SP-NFT
**Simulated SP–NFT training scheme**						
Landers et al., 1991	Pre elite archers (24)	Increase left hemisphere SCP activity (T3)	Move a horizontal bars on computer	1	30 min	Visual
Gong et al., 2020	Amateur Shooters (45)	Increase left hemisphere alpha activity (T3)	Try to make the image clear and the music louder.	6	25 min	Visual-Auditory
**Attention-focusing training scheme**						
Paul et al., 2011	University level archery (24)	Enhance SMR, inhibit theta and bete2 (Cz)	Keep the animation moving	12	20 min	Visual-Auditory
Faridnia et al., 2012	National swimmers (20)	(1) Increase beta1 and SMR, decrease theta and bete2; (2) increase beta, decrease beta2 (C3,C4).	Play a video game	12	40 min	Visual
Rostami et al., 2012	National and provincial rifle shooters (24)	(1) Increase SMR, decrease bete2 (C, C4); (2) Increase crossover between alpha and theta, decrease beta2 (Pz).	Play a computer game	15	60 min	Visual-Auditory
Mikicin et al., 2015	Student athletes (35)	Increase beta1 and SMR, decrease theta and beta2 (C3, C4).	Complete a placing balls computer game.	20	30 min	Visual-Auditory
Gong et al., 2020	Amateur Shooters (45)	Increase SMR (C3,Cz,C4).	Try to make the image clear and the music louder.	6	25 min	Visual-Auditory
**Relaxation training scheme**						
Raymond et al., 2005	University level dancers (24)	Increase theta and decrease alpha (Pz)	Imagine dancing	10	20 min	Auditory
Gruzelier, 2014	University level dancers (64)	Increase theta and decrease alpha (Pz)	Imagine dancing	10	20 min	Auditory
**Monitoring-guided training scheme**						
Arns et al., 2008	Amateur golfers (6)	Personalized event-locked EEG Profiles (FPz)	Golf putting task	3	N/A	Auditory
Kao et al., 2014	Elite golfers (3)	Decrease theta (Fz)	Golf putting task	1	25 min	Visual-Auditory
Ring et al., 2015	Recreational Golfers (24)	Reduce theta and high alpha power (Fz)	Golf putting task	3	60 min	Auditory
Cheng et al., 2015a	Pre-elite and elite golfers (16)	Increase SMR (Cz)	Golf putting task	8	30-45 min	auditory

### Simulated Training Scheme

Simulated SP–NFT training scheme is feedback training that simulates the dynamic changes of brain activity during real exercise. This scheme firstly identifies the neural markers of sport performance through feature extraction and statistical analysis. Next, the neural markers are selected as feedback features and trained in the laboratory according to traditional visual or auditory NFT. The participants are instructed to recall their exercise scene and regulate this feedback feature during NFT. This method is similar to traditional motor imagery training: athletes can recall the actual sport process by using feedback images or music, imagine their movement details and body feelings during this process, and thus improve their own performance.

Simulated SP–NFT records participants’ brain state when they achieve their best sport performance during the actual exercise and also screens out the reliable and stable brain nerve characteristics that can reflect that performance. Thereafter, participants are asked to perform SP–NFT in a quiet and comfortable environment and instructed to recall a state that participant achieve the best sport performance. For example, instructing the shooter recall the memory of achieving 10 scores during a recent training or competition. Through SP–NFT, participants can “return” to the brain nerve state that acquires the best sport performance.

This scheme uses SP–NFT to help participants learn the process of achieving their best sport performance, allowing them to more easily reach their best competitive state and thus improving their performance. However, an important precondition exists for this scheme, i.e., feedback characteristics must match the brain state that the participant obtains during his or her best sport performance. Because of individual differences, the neural state of each participant will not be precisely the same as another participant when achieving that performance. If the selected feedback characteristics cannot reflect the best performance, then this training has no effect and can even produce an opposite training effect.

For example, although Landers et al. (1991), Gong et al. (2020) selected similar SP–NFT schemes, they obtained different experimental results due to participants belonging to different levels of expertise. Landers et al. (1991) found that inhibiting brain activity in the left temporal region effectively improved the shooting performance of professional archery athletes (Landers et al., 1991). However, Gong et al. (2020) showed that an SP–NFT inhibiting brain activity in the left temporal region does not significantly improve the performance of amateur shooters, and many participants even showed decreased performance (Gong et al., 2020). Importantly, the phenomenon of left temporal activity inhibition was detected by analyzing EEG data of expert shooting preparation stages, which may not be suitable for amateur shooters. As a result, if both experts and amateur athletes adopted the same simulated SP-NFT, different training results may be obtained.

These varying results may be because the inter-individual differences in motor level led to large differences in the state of the brain nerve when they obtained their best performance, so that the effective SP–NFT for expert shooters is not applicable for amateur shooters (Hatfield et al., 1984; Bertollo et al., 2016; Gong et al., 2019). As a result, the simulated SP–NFT requires researchers to confirm whether the feedback characteristics match the brain state during the athletes’ best performance before training. For example, in shooting, each participant’s EEG signal characteristics can be obtained by analyzing the difference between high and low scores. These EEG characteristics are selected as the participant’s SP–NFT. That is, the best case is based on the personalized feedback feature for each participant.

This scheme is currently used only in closed (i.e., usually individually performed) sports such as shooting and archery, because the brain activity in these sports can be measured accurately so as to extract relatively stable brain nerve characteristics. For open sports (i.e., usually team-performed sports), such as football, basketball, and hockey, few studies have adopted a simulated SP–NFT scheme to improve performance.

However, this lack of research does not mean that this scheme is ineffective for open sports. Previous studies have observed that highly similar brain nerve activities exist between actual exercise and motor imagery. Athletes who watch videos of the sport process have brain activity similar to performing the actual sport (Babiloni et al., 2009; Munzert et al., 2009; Hétu et al., 2013). These studies provide a potentially viable solution for simulated SP–NFT. Participants’ brain activity during motor imagery or watching motor videos are collected, and their neural features related to best performance are extracted as feedback signals. Afterward, simulated SP–NFT is performed on the participants.

### Attention-Focusing Training Scheme

Attention-focusing SP–NFT schemes are some of the most widely used in current research. This scheme’s principle is to train participants’ attention in order to enhance their attention level and enable them more focused in the course of exercise for a longer time, so that they can achieve a relaxed yet focused, alert, and ready state. The researchers believe that this state is beneficial to participants’ behavioral control, and if it can be achieved in conditions of actual sport performance, sport performance will be improved (Ros et al., 2009; Collura, 2013).

The attention-focusing scheme usually adopts the method of reducing the theta rhythm power or increasing the SMR or beta1 rhythm power. The training electrode is usually set in the central region position, such as C3, Cz, and C4, and the training feature could be either a single frequency of a single electrode or the multiple frequencies of multiple electrodes. Reduction of the power of theta rhythm can inhibit the drowsiness, fatigue, and distraction, and an increase of SMR and beta1 will make participants more focused and pay attention. Finally, the participants can reach a quiet, smooth, and focused state of mind (Harmison, 2006).

Attention-focusing training has the advantage of a clear physiological mechanism, and many studies have repeatedly proved the close relationship between the characteristics of the frequency band (mainly SMR and beta1) and the physiological state used in this scheme (Vernon et al., 2004; Marzbani et al., 2016). Therefore, the trainability of this scheme need not be verified, and most participants can learn and train smoothly.

Attention-focusing SP–NFT can be applied to most closed sports, as the quiet and focused state caused by this training is beneficial. Participants can pay attention to their own motion and avoid unnecessary interference in their thinking, so the completion of the action is more efficient and smooth.

Many cases exist for the application of the attention-focusing SP–NFT scheme. Paul et al. (2011) successfully improved shooting performance in university-level archery shooters by this scheme (Paul et al., 2011). Likewise, Rostami et al. (2012) enhanced expert shooters’ performance using this scheme (Rostami et al., 2012).

In addition, attention-focusing training can be applied to open sports. In general, attention enhancement may be beneficial for doing any movements, not just fine movements. Although increasing attention may not directly improve sport performance, SP–NFT may affect participants’ daily training and learning, which can indirectly improve their sport performance by improving learning efficiency. For example, Faridnia et al. (2012) trained expert female swimmers and reduced their pre-competition anxiety. Mikicin et al. (2015) trained 35 student athletes and found that their speed, effectiveness, and work accuracy improved (Mikicin et al., 2015).

### Relaxation Training Scheme

Relaxation training is an effective SP–NFT scheme that regulates mood and relieves stress. This method inhibits the alpha rhythm and enhances the theta rhythm of the parietal region position (Pz). Through this scheme, participants can enter a deep “silence” state in which they completely relax their spirit and effectively reduce their anxiety. At the same time, participants in this state can also guide themselves to fully recall the details and their feelings during the competition training and sometimes can even induce themselves to produce some “inspiration” or creative thinking (Gruzelier, 2009, 2014b).

The purpose of this training method is exactly the opposite of the attention-focusing scheme. The relaxation training scheme increases theta rhythm and inhibits high frequency components (mainly alpha rhythm). Therefore, relaxation training requires that participants do not concentrate so as to relax their spirit.

At present, relaxation training SP–NFT is used mainly to improve dance performance (Raymond et al., 2005; Gruzelier et al., 2014). Therefore, we hypothesis this training scheme may also benefit sports such as rhythmic gymnastics, figure skating, and diving, which require some creative thinking (Gruzelier, 2014b). Because of the weak antagonism of these sports, the relaxation SP–NFT can reduce participants’ pre-competition anxiety and improve their competitive state during competition, ultimately improving performance.

### Monitoring-Guided Training Scheme

Monitoring-guided SP–NFT has developed rapidly in recent years. Because of its short experimental period and quick effect, this scheme overcomes the traditional SP–NFT shortcoming of being time intensive. The principle of monitoring-guided SP–NFT is the Yerkes–Dodson theory–the inverted-U relationship between arousal degree and sport performance (Yerkes and Dodson, 1908). This approach can lead participants to their best arousal level matching their best sport performance, then allowing participants to perform the sport action at their best arousal level or to continue to be in their best arousal state during the whole action process. To improve sport performance, one might reach the best individual arousal level.

The biggest feature of monitoring-guided SP–NFT, which is different from traditional SP–NFT, is its close combination with training action, that is, feedback training and exercise processes are carried out synchronously. Kao et al. (2014), Cheng et al. (2015a) used this feedback approach during golfers’ putting. This approach monitors participants’ brain activity in the preparation stage of putting, giving them real-time audio feedback. A loud sound indicates that their current state is not good, and a soft sound indicates that their current state is good. Participants choose the appropriate putting time according to the guidance of sound. This feedback training scheme has achieved better training results and has significantly improved putting performance (Kao et al., 2014; Cheng et al., 2015a).

Faller et al. (2019) conducted a monitoring-guided SP–NFT experiment and found that this training can effectively regulate the arousal degree and maintain the arousal degree of pilots at their best levels. The training also improved pilot performance in a boundary avoidance task (Faller et al., 2019).

For many years, one important reason why traditional SP–NFT advanced slowly in the field of sport training is that the training cycle is too long. Traditional SP–NFT usually requires 5–10 sessions before post-test experiments can be performed to test effectiveness (Gruzelier et al., 2006; Hammond, 2011). Many shortcomings exist in traditional long training schemes. From the experimenter’s point of view, the workload is large. From the participants’ point of view, the long training brings fatigue. Some participants may have other interference factors such as living far away from the experimental site, family or other emergencies, and other inabilities to persist with the long training. Because of the lack of timely feedback on their sport performance, participants have doubts about whether SP–NFT can actually improve their performance. Training enthusiasm and confidence may gradually decrease with the increase in training time.

Monitoring-guided SP–NFT combines the feedback training and the actual action processes, which could provide feedback in real-time and test the effects of that feedback by analysis of the completion of the target task in time. This method solves the problem that the training time in traditional SP–NFT is too long. Monitoring-guided SP–NFT experiments are usually completed within 1 h, which can not only carry out the SP–NFT research but can also test the training effect (Kao et al., 2014; Cheng et al., 2015a; Ring et al., 2015; Faller et al., 2019). No pre-test and post-test saves a great deal of experimental time. On the basis of this characteristic, monitoring-guided SP–NFT is likely to have the most potential to be used commercially in the short and long term.

At present, the feedback effect of the reported monitoring-guided SP–NFT is short-term, and no research has been carried out on training effects on long-term sport performance. Although SP–NFT only reported short-term effects, this training technique still has important applications. On the one hand, SP–NFT can be used as a neurodoping technique to warm up before a game, allowing athletes to improve their competitive state before the game. On the other hand, SP–NFT can also be used as a special auxiliary training method in daily training to improve the learning efficiency of participants’ motor skills (Ring et al., 2015). Certainly, whether NFT has long-term effects remains a relevant research question.

### Classification Methods and Advantages

Combined with the user’s feelings and feedback characteristics, the SP–NFT classification methods based on user experiences are as follows: (1) Simulated SP–NFT is similar to imagery training in the laboratory. The feedback feature corresponds to the related neural biomarkers during the course of exercise. (2) Attention-focusing SP–NFT requires participants to focus on a specific event or game. The feedback features aim mainly to reduce theta activities and improve SMR or Beta1 activities. (3) Relaxation SP–NFT requires participants to relax as much as possible, reducing mental anxiety, whereas the feedback features aim mainly to increase theta and reduce alpha activity. (4) Monitoring-guided SP–NFT is an auxiliary means in the process of movement, and the feedback features need to be determined according to the neural biomarkers in the process of movement completion.

For users, the advantage of this classification is to enable them to quickly understand what SP–NFT can do and which needs it can meet and to help coaches or athletes quickly choose their own training programs according to their needs. For example, if you want to recall how you feel when you perform a real movement, you can choose a simulated SP–NFT, and if you want to improve your concentration in competition, you can choose an attention-focusing SP–NFT.

For researchers, the advantages of this classification are the ease of comparison of SP–NFT with other similar methods and the quantitative analysis of SP–NFT advantages. For example, researchers can compare the training effect of attention-focusing SP–NFT with the traditional “needle piercing” training method (a traditional Chinese training method to increase shooters’ attention), and test the difference in the effectiveness of the two methods in improving attention. As for relaxation SP-NFT, it can be compared with traditional listening to music or meditation as relaxation methods to test which relaxation is more effective.

## Some Issues in SP–NFT Development

While some progress has been made in SP–NFT research, some important but unsolved issues are affecting the further development of SP–NFT technology, and clarification of these issues can help promote its research.

### Mechanism of Sport Performance Improvement

SP–NFT could change the brain’s neuroplasticity by operant conditioning, regulating the activation of brain regions and the neural pathways between brain regions. But why can this change improve sport performance, and will this change affect other aspects of participant behavior? These are the basic theoretical questions.

SP–NFT mechanisms behind the action may be explained from two perspectives. First, from reinforcement learning, SP–NFT allows participants to repeat the return to the state of the brain during actual exercise or to repeat exercising the brain regions related to motor control function. This training scheme allows participants to control the details of their movements more effectively, thus improving their performance. Similar to the noted AlphaGo, which by learning from tens of thousands of experts in their own games to become an invincible Go board game champion, participants can eventually improve their performance (Silver et al., 2016).

On the other hand, the mechanism of SP–NFT can be explained from the perspective of improving basic ability, which mentioned here is not strength, endurance, coordination, or other physical qualities but instead refers to the brain’s ability to attend to attention, volition, relaxation, and other mental skills. SP–NFT has not only improved participants’ state (e.g., their attention, anxiety) after training (Mirifar et al., 2017), but participants have also mastered their ability to self-regulate their mental state. Just as people with strong self-control are more likely to succeed, those who have mastered the ability to regulate their own mental state may be more likely to achieve excellence than those who cannot. In addition, learning to self-regulate mental state cannot only support participants in sport but also in other aspects of their lives as well (Collura, 2013).

Measuring multiple dimensional data will help further reveal the underlying successful mechanisms behind SP–NFT. In most studies, researchers were mainly concerned about whether participants’ sport performance has been improved, so most have measured only performance in the pre-test and post-tests, not paying attention to other aspects.

However, Mikicin et al. (2015) provided a better demonstration of the multiple dimensional measures taken in the SP–NFT, including participant attention response, Kraepelin work curves, and other indicators, and found that SP–NFT can also improve cognitive function (Mikicin et al., 2015). Other studies have reported changes in other aspects such as EEG characteristics during feedback training or resting state, indicating the neuroplasticity of SP–NFT (Gong et al., 2019). The more diverse the data reported, the more helpful it is to fully reveal the relevant mechanisms of SP–NFT. Therefore, in future studies, researchers should report data other than sport performance.

### Feedback Scheme and Feature Selection

Before using SP–NFT, participants should be evaluated comprehensively and accurately. Experimenters should fully consider participants’ conditions and their goals for training so as to determine which SP–NFT scheme best suits them.

First, the purpose of training needs to be considered, which is determined from full communication with coaches or participants. Do participants want to improve their attention? Ease their anxiety? Or find their best sport state? Different problems have different targeted SP–NFT solutions, and the selection of the incorrect SP–NFT is likely to lead to the failure of training.

Second, it is necessary to consider participants’ skill levels, because the skill levels of the participants may impact the effect of SP-NFT. Gong et al. (2020) inferred that highly skilled shooters improved their performance using a simulated training scheme, while amateur shooters were better served by an attention-focusing scheme (Gong et al., 2020). Moreover, Ring et al. (2015) trained theta and alpha2 reduction in golf amateurs’ (simulating the brain pattern in the expert putting preparation stage) by a monitoring-guided training scheme, but found no significant training differences between the experimental and control groups (Ring et al., 2015). However, Kao et al. (2014), Cheng et al. (2015a) trained expert athletes using monitoring-guided SP–NFT according to similar principles with improved putting performance (Kao et al., 2014; Cheng et al., 2015a).

It is expected that experts are more skilled and have better control over their motor behavior, so they can easily refocus their minds back to the state of exercise execution and maintain a highly similar state to the real exercise. However, finding a state similar to real sport is difficult for amateurs and ordinary sport enthusiasts. Even if amateur athletes learn this way of regulating their own brain state, they may not be able to improve their sports performance.

Finally, it is necessary to consider the degree of SP–NFT acceptance and the ability of the brain to be regulated, which should be judged by a certain number of pre-experiments. The feedback feature determines the SP–NFT training scheme, but the opposite is not true. For example, enhancement of C3 SMR rhythm and the reduction of theta rhythm are both attention enhancement schemes, but whether to choose to enhance the SMR or reduce the theta is also an important issue to be discussed and analyzed. Some participants found it easier to reduce theta, while enhancing SMR was difficult. Therefore, it is necessary for coaches to choose the appropriate SP–NFT scheme. A feasible scheme is to first carry out simple training, then gradually increase training difficulty. If the initial scheme is too difficult, participants’ confidence is likely to be adversely affected, resulting in a quick abandonment of training or having doubts about themselves.

### Basis of NFT Learning Theory

Researchers who want to develop or test a new SP–NFT scheme should first test the theoretical basis of that scheme before testing the training effect. Although SP–NFT shares a solid learning theory with operant conditioning theory, it still is important to test the trainability of a new scheme.

A test of trainability includes verifying whether participants can change the feedback characteristics used in the SP–NFT. In SP–NFT, it is theoretically possible to use any EEG characteristics as feedback characteristics, such as enhancing the activity of a certain brain region, enhancing the connectivity between the two brain regions, and even activating the amygdala of the brain using LORETA technology (Thatcher et al., 2020).

Multiple feedback training goals can also be set at the same time, such as requiring participants to reduce theta and beta2 rhythms while enhancing SMR (Paul et al., 2011). The diversity of SP–NFT leads to different degrees of difficulty between different schemes. Although some feedback characteristics are related to behavioral performance, they are not necessarily a suitable training goal. Participants may need to spend much energy learning to control feedback characteristics. Choosing inappropriate feedback characteristics may lead to the failure of the experimental results.

Another aspect of trainability is to test whether SP–NFT can change participants’ baseline neural characteristics and produce neuroplasticity, an important basis for proving that SP–NFT affects behavior by changing brain activity (Cho et al., 2007; Zoefel et al., 2010; Enriquez-Geppert et al., 2014). For example, many researchers have studied the trainability of SMR and theta characteristics. Researchers who want to study a new feedback characteristic should first pay attention to the trainability test.

### Training Type and Parameter Selection

Before conducting an SP–NFT study, feedback type, and parameter selection must be considered. Training type mainly refers to the choice of visual or auditory feedback, or a combination of the two. Parameter selection is the training time in each training course, the number of training sessions, and the strategies for avoiding boredom or weariness.

NFT mainly includes visual, auditory, and tactile feedback, among which visual and auditory are the most commonly used. A combination of visual and auditory feedback has been reported to be more effective than a single type of feedback, but this conclusion may be applicable only to a particular SP–NFT scheme and has not been widely validated (Vernon et al., 2004). Some researchers believe that the presence of both visual and auditory feedback may be affected by mutual interference. In addition, if the two feedback modes are not well combined, they may easily confuse the participants and adversely affect the feedback effect. Researchers who support using both types of feedback believe that the combination of the two allows participants to inadvertently ignore one feedback and rely on the other to remind them to continue to train (Lal et al., 1998; Vernon et al., 2004).

Moreover, the SP–NFT training process can be combined with the exercise process to achieve better training results. For example, Cheng et al. (2015a) studied improving golfers’ sport performance by monitoring-guided SP–NFT, which used individual auditory feedback (Cheng et al., 2015a). Using this example, it is believed that the visual function of the human body is usually occupied in the actual motion, so the auditory feedback may have greater application potential in SP–NFT.

Before the SP–NFT is carried out, specific feedback parameters need to be set, such as the criteria for determining rewards, the duration of training and relaxation, the duration of one session of feedback, and the duration of the whole course of feedback. These parameters have an important effect on the training effect. In the judgment standard of reward, it is best to follow the principle of “easy first, then difficult” to avoid reducing participant confidence and interest. The duration of each training session is usually 20–30 mins; the duration of the entire course can be determined by participants’ feelings and the purpose of the research.

In previous research, the number of sessions has ranged from 1 to 40. Some researchers believe that, to detect significant physiological changes in the brain, 5–10 sessions of training are required at least (Hammond, 2011).

Although some studies have used fewer feedback sessions, they still report positive training results. Therefore, the optimal number of training sessions in SP–NFT, the minimum number of nodes that produce physiological changes and changes in sports performance, and the relationship between physiological changes and changes in sport performance are still important issues to explore.

### Rigorous and Reproducible Research

In the medical field, before carrying out a clinical intervention study, researchers should set up both an experimental and a placebo-control group and use a double-blind or triple-blind scheme to ensure rigor and effectiveness. For SP–NFT studies, researchers often use this experimental method to test the effectiveness of training.

Because experimental participants are easy to recruit and the experimental risks are small in NFT studies in the field of healthy cognitive science, the experimenter can usually choose college students, and a double-blind placebo-controlled design can be adopted. However, in sport science, if the object of the experiment is top athletes, such an experimental design may be problematic.

First, the number of athletes willing to participate in the study may be small, the participants may be difficult to recruit due to schedules, the number of top athletes in a sport may be only a dozen or dozens, and the number of participants willing to participate in the study may be even smaller. The placebo-control group usually uses false and ineffective SP–NFT, which not only occupies the athletes’ valuable training time but may even affect the athletes’ mood because of persistent feedback of failure (e.g., random signals or EEG signals from other participants), which leads to a decline in performance.

Because of these problems, only a few SP–NFT studies have adopted a strict double-blind placebo-control design, and most studies have used only passive control groups or even no control groups. Compared with the active control group, the passive control group does not have any SP–NFT, so the rigor of the study is greatly reduced for the whole experiment.

The solution to this problem may include expanding the screening range of experimental participants, that is, the screening range is not limited to top sports experts but also includes amateur athletes or even sport enthusiasts. Also, it may be helpful to shorten the training sessions and the time between post-test experiment and feedback training, so as to test the effect of the feedback training in time and reduce the time occupied by the entire research project. Finally, adjustment of the control group setting (i.e., not using the SP–NFT-based placebo-control group but other traditional training methods such as meditation and mindfulness training) is also feasible. This experimental design may be more in line with the realities of conducting SP–NFT research.

### Non-specificity Issues

The EEG-based NFT is a more macro brain regulation than drug intervention, so its specificity is not very good (i.e., after training, participants may experience changes beyond the training goals).

Gruzelier (2014c) have summed up the NFT specificity in three aspects: (1) the specificity of feedback effect (Outcome specificity), (2) the specificity of feedback frequency band (Frequency band specificity), and (3) the specificity of feedback position (Topographical specificity) (Gruzelier, 2014c). The non-specificity of the feedback effect is the additional changes in the non-training target caused by the SP–NFT. For example, after SP–NFT, participants were found to have changes in attention, working memory, and other aspects in addition to motor performance (Paul et al., 2011; Faridnia et al., 2012).

SP–NFT changes to the non-target frequency band and the non-target position are also known as the “entrainment” effect, which usually occurs in the frequency band and position near the target frequency band and the target position (Collura, 2013). For example, a training goal is to enhance alpha rhythms, but theta and beta1 rhythms are also found to be enhanced. Training position is Cz, but the results found that the FCz and CPz nearby location characteristics have also changed.

Whether SP–NFT has long-term effects on exercise is still a question under exploration because the current SP–NFT studies have only reported short-term results after training and did not perform follow-up studies for investigation of long-term effects. However, in the field of NFT for ADHD intervention, there are some inconclusive findings about the long-term specificity of NFT, which have been reported in a recent meta-analysis (Van Doren et al., 2019). Therefore, we speculate that in the field of sports, SP–NFT may also not lead to long-term specific changes.

Overall, because of SP–NFT’s non-specificity in feedback effect, some researchers believe that SP–NFT should be used with caution. Similar to the reasoning for the use of clinical trials to detect the side effects of drugs, it should be clear that participants may have adverse reactions after SP–NFT.

## Future Directions of SP–NFT Development

### SP–NFT Based on New EEG Characteristics

Traditional SP–NFT most often uses EEG frequency band power characteristics. Many studies have shown good outcomes from using frequency band power as a feedback feature and have developed a series of widely used SP–NFT schemes. With the development of EEG signal-processing technology, scholars have continuously extracted new characteristics from EEG, which can also be applied in SP–NFT in the future.

(1) EEG characteristics of non-traditional frequency bands

Traditional EEG characteristics include SCP, theta, alpha, SMR, and beta1 and beta2 rhythms. NFT studies in recent years have extended the non-traditional EEG such as gamma frequency band and infra-slow potentials. With the development of this technology, scholars have gradually begun to detect frequency components outside the classical frequency bands. For example, some researchers have found that gamma frequency band characteristics are associated with activities that require high concentration, such as meditation and mindfulness (Fabio et al., 2013; Schoenberg and Speckens, 2015). Therefore, SP–NFT based on non-traditional frequency band EEG characteristics also has great potential.

(2) Functional connectivity and brain network characteristics

Functional connectivity and brain networks have developed rapidly in EEG research. Many studies have found that a close relationship exists between resting brain functional connectivity and brain network characteristics and cognitive or sport performance (Zhou et al., 2012; Gong et al., 2017). Compared with traditional single-brain region activation and inhibition training, this method pays more attention to the information interaction and overall coordination of each region. At present, some studies have gradually begun to study NFT based on functional connectivity and brain network characteristics and have obtained preliminary significant results (Ramot et al., 2017; Thatcher et al., 2020). In the future, SP–NFT based on functional connectivity and brain networks may be an important training scheme.

(3) EEG traceability characteristics

Neurofeedback training based on the traceability algorithm LORETA is a training method that can trace electrical signals on the brain’s surface to the active area inside the brain and then uses the NFT to train a specific brain area directly, which can achieve a training goal that traditional NFT cannot (Congedo et al., 2004). The advantage of LORETA-based SP–NFT is that this training is more targeted than traditional SP–NFT, which greatly improves the spatial resolution of SP–NFT and greatly increases the scope of training.

However, the mathematical model of the LORETA algorithm is fixed, and many kinds of neural activities in the brain can cause similar electrical activities in the scalp. The solution obtained by the traceability algorithm is only one of many possible solutions. Meanwhile, LORETA NFT can train participants only to activate the target brain area but not to “relax” that area. Compared with the “enhancing and decreasing” of the traditional EEG frequency band NFT scheme, the directionality of the LORETA NFT is reduced. Today, LORETA-based SP–NFT has not been tried in the sport science, but with the continuous development of technology, the application scenarios of SP–NFT based on LORETA will continue to increase.

Beside the three schemes mentioned above, Z score NFT, flash stimulus NFT, and other methods can be used (Collura, 2013; Lucas, 2015). Although preliminary studies of these methods have appeared in fields other than sports science, they have not been reported in SP–NFT. These methods may also have application potential in sport science.

### SP–NFT Based on Multi-Modal Neural Signal Characteristics Fusion

Multi-modal signal fusion acquisition, an important research area in neuroscience, is divided into multi-modal signals with different technologies and multi-modal signals with similar technologies. The typical application of the former is the synchronous acquisition of different neuroimaging techniques, such as EEG–fNIRS or EEG–fMRI, while the typical application of the latter is the synchronous acquisition of similar physiological and electrical techniques, such as EEG, electro-oculography (EOG), and electromyogram (EMG). This section mainly discusses the latter technology.

Multi-modal SP–NFT is a biofeedback method that integrates EEG, EOG, and EMG. Strictly speaking, multi-modal SP–NFT is no longer a type of NFT, but the purpose of this feedback training is still the same as that of traditional NFT in that it helps participants regulate their own state, thus improving their own mental condition or their external performance. In clinical medicine, Maurizio et al. used feedback training combined with EEG and EMG signals to treat children with ADHD and achieved good results (Maurizio et al., 2014).

Multi-modal acquisition technology can synchronously collect physiological data of multiple dimensions, such as EEG, EMG, and EOG, through data analysis and characteristic extraction; real-time monitoring; and analysis of the brain, muscle, and visual functions related to motor behavior, which are involved mainly in actual motor behavior. Using this prior knowledge, participants are instructed to improve their own state and their sport performance.

For example, in SP–NFT for rifle shooting, the state of brain nerve and forearm muscle (i.e., control of the trigger process) can be measured synchronously, and then feedback can be provided to participants by extracting the corresponding indexes in real-time. This process can not only train the brain nerve state in the shooting preparation, but it also can further train the fine movement of the muscle when pulling the trigger. Multi-modal SP–NFT may benefit coaches and athletes, making it important for research development.

### SP–NFT Based on New Brain Image Technology

Traditional SP–NFT is based mainly on EEG technology, but with the rapid development of neuroimaging and computer technologies, these are increasingly used in NFT. These technologies are based mostly on fMRI and fNIRS NFT. The principle of these two feedback methods is similar, which is based on neuroimaging technology to activate the target brain region to achieve the purpose of training and improve external performance.

The advantage of fMRI-based NFT techniques is that this technology has a high spatial resolution, so it can be trained to almost all locations in the brain, even regions such as the hippocampus and amygdala that are difficult to locate by other methods (Zotev et al., 2011). At present, fMRI-based NFT is also used in the treatment of mental illness and emotional regulation (Pavla et al., 2019). However, the shortcomings of fMRI include high cost and low time resolution. Therefore, the fMRI-based SP–NFT is still limited to functional verification, and the prospect of popularization is low.

Compared with EEG, fNIRS is more powerful against motor artifacts and therefore, more suitable to study the neuronal mechanisms in real motor behavior (Seidel-Marzi and Ragert, 2020). Many previous studies have successfully applied fNIRS to study functional brain adaptations during real sports such as playing table tennis, cycling, and climbing (Balardin et al., 2017; Seidel et al., 2019; Carius et al., 2020), and obtained results difficult to obtain with other technologies. Meanwhile, although fNIRS-based NFT has been applied to emotional regulation and some neurological diseases (Aranyi et al., 2015; Blume et al., 2020; Rieke et al., 2020), reports on their application to promoting sports performance are relatively rare. Therefore, we look forward to the further development of fNIRS-based SP–NFT and the further expansion of SP–NFT from the laboratory to outdoor sports.

A novel SP–NFT research idea is to combine these methods. fMRI and fNIRS techniques are used to determine the brain function area, which is closely related to the performance of a certain exercise. These key areas can be found by using fMRI during motor imagery or watching a sports video or even can be directly detected in real exercise situations using fNIRS. Recent studies by Rieke et al. (2020) have fully combined the advantages of fMRI and fNIRS techniques to develop an SP–NFT system for motor rehabilitation of stroke patients, which has achieved better training results than traditional feedback and can be applied to SP–NFT in the future (Rieke et al., 2020).

### SP–NFT Combining Novel Interactive Technologies

Conventional NFT is usually based on commercial software or systems developed independently. The feedback interface is relatively simple and lacks interesting incentive strategies or customized designs. The monotony of the feedback form may cause participants to lose interest in training, meaning they are not completely immersed in the training environment and cannot achieve the ideal training effect.

One way to solve this problem is to combine NFT technology with a variety of human–computer interaction technologies to construct an “immersion” training environment for better experiences. For example, virtual reality (VR), augmented reality (AR), and mixed reality technologies can greatly improve participants’ sensory experiences.

Some studies have carried out EEG experiments in VR/AR environments, such as those studying emotion recognition and assessing fatigue, and have achieved good results (Martin et al., 2013; Slobounov et al., 2013; Rodríguez et al., 2015). Other studies have combined VR and NFT techniques and applied them to the treatment and rehabilitation of nervous system diseases such as stroke and Alzheimer’s disease. Using these techniques can enhance the therapeutic effects of traditional NFT (Kober et al., 2016; Federica et al., 2020).

Although virtual interaction has not yet been seen in SP–NFT, it can be expected that the combination of the two technologies will make brain regulation more effective and help participants improve their sports performance. For example, Baqapuri et al. (2021) found that the participants were engaged within the VR environment, improving their game performance over time while simultaneously employing NFT strategies. The study also pointed out that VR games can effectively slow down the reduction of participants’ training interest and maintain the NFT training effect (Baqapuri et al., 2021).

It is important to consider that the introduction of VR techniques into EEG- or fMRI-based NFT is limited by these neural signal acquisition techniques. If participants have large body movements during the feedback period, the collected signal contains motion artifacts that affect the feedback effect. Therefore, VR techniques in EEG or fMRI NFT training may only help subjects obtain better immersive environment. If participants want to move or perform a real motion task in a VR environment, fNIRS-based SP–NFT may be a better method.

### Application and Commercialization of SP–NFT

While NFT has been widely used mostly in clinical medicine, SP–NFT has not been widely popularized in sport science. Reasons may include a long preparation time before training and training duration, participants not being comfortable wearing electrodes, the equipment interfering with normal athletic training, the improvement of performance not being significant enough, and the training generally not being attractive to users. As a result, many scholars are concerned about when SP–NFT technology can move from the laboratory and into practical commercial use.

One promising commercial SP–NFT option is monitoring-guided SP–NFT. The biggest advantage of this scheme is that it can greatly shorten the test time for training effect evaluation, so participants can understand more quickly the effects of SP–NFT. If the effects are good, participants will prefer and be more willing to accept this form of training.

An important problem that restricts SP–NFT commercialization is the long preparation time before training. Before training begins, it is necessary to install the EEG cap, apply the electrode gel, adjust electrode impedance, and so on. Dry electrode technology, wireless data transmission technology, and customized EEG cap technology have made great contributions toward solving this problem. Some studies have shown that the performance of dry electrode technology is closer to the traditional electrode, and good experimental results have been obtained (Collado-Mateo et al., 2015).

An SP–NFT feedback system based on these new technologies could further expand training application scenarios. For example, by using dry electrode and radio electrode technology, athletes can configure their own training programs and complete them at any time and place; this personalization would have great potential commercial value and support the wide use of SP–NFT (Kam et al., 2019).

## Conclusion

SP–NFT is an important research direction in the field of neurofeedback. To promote the further development of this research, this article proposes a new method for SP–NFT classification from the perspective of user experience. Through the classification and discussion of various SP–NFT schemes, applicable scenarios, application effects, and technical characteristics are detailed. On the basis of the SP–NFT application reported in the current literature, the article also summarizes key problems in current SP–NFT development and looks forward to future development directions. This information can also help researchers in related fields sort out research ideas and can provide a valuable reference for finding new research paths.

## Author Contributions

All authors listed have made a substantial, direct and intellectual contribution to the work, and approved it for publication.

## Conflict of Interest

The authors declare that the research was conducted in the absence of any commercial or financial relationships that could be construed as a potential conflict of interest.
